# Validity and Feasibility of the Monitoring and Modeling Family Eating Dynamics System to Automatically Detect In-field Family Eating Behavior: Observational Study

**DOI:** 10.2196/30211

**Published:** 2022-02-18

**Authors:** Brooke Marie Bell, Ridwan Alam, Abu Sayeed Mondol, Meiyi Ma, Ifat Afrin Emi, Sarah Masud Preum, Kayla de la Haye, John A Stankovic, John Lach, Donna Spruijt-Metz

**Affiliations:** 1 Department of Chronic Disease Epidemiology School of Public Health Yale University New Haven, CT United States; 2 Department of Population and Public Health Sciences Keck School of Medicine University of Southern California Los Angeles, CA United States; 3 Research Laboratory of Electronics Massachusetts Institute of Technology Cambridge, MA United States; 4 Department of Electrical and Computer Engineering School of Engineering and Applied Science University of Virginia Charlottesville, VA United States; 5 Department of Computer Science School of Engineering and Applied Science University of Virginia Charlottesville, VA United States; 6 Department of Computer Science School of Engineering Vanderbilt University Nashville, TN United States; 7 Department of Computer Science Dartmouth College Hanover, NH United States; 8 School of Engineering and Applied Science The George Washington University Washington, DC United States; 9 Center for Economic and Social Research Dornsife College of Letters, Arts, and Sciences University of Southern California Los Angeles, CA United States; 10 Department of Psychology Dornsife College of Letters, Arts, and Sciences University of Southern California Los Angeles, CA United States

**Keywords:** ecological momentary assessment, wearable sensors, automatic dietary assessment, eating behavior, eating context, smartwatch, mobile phone

## Abstract

**Background:**

The field of dietary assessment has a long history, marked by both controversies and advances. Emerging technologies may be a potential solution to address the limitations of self-report dietary assessment methods. The Monitoring and Modeling Family Eating Dynamics (M2FED) study uses wrist-worn smartwatches to automatically detect real-time eating activity in the field. The ecological momentary assessment (EMA) methodology was also used to confirm whether eating occurred (ie, ground truth) and to measure other contextual information, including positive and negative affect, hunger, satiety, mindful eating, and social context.

**Objective:**

This study aims to report on participant compliance (feasibility) to the 2 distinct EMA protocols of the M2FED study (hourly time-triggered and eating event–triggered assessments) and on the performance (validity) of the smartwatch algorithm in automatically detecting eating events in a family-based study.

**Methods:**

In all, 20 families (58 participants) participated in the 2-week, observational, M2FED study. All participants wore a smartwatch on their dominant hand and responded to time-triggered and eating event–triggered mobile questionnaires via EMA while at home. Compliance to EMA was calculated overall, for hourly time-triggered mobile questionnaires, and for eating event–triggered mobile questionnaires. The predictors of compliance were determined using a logistic regression model. The number of true and false positive eating events was calculated, as well as the precision of the smartwatch algorithm. The Mann-Whitney *U* test, Kruskal-Wallis test, and Spearman rank correlation were used to determine whether there were differences in the detection of eating events by participant age, gender, family role, and height.

**Results:**

The overall compliance rate across the 20 deployments was 89.26% (3723/4171) for all EMAs, 89.7% (3328/3710) for time-triggered EMAs, and 85.7% (395/461) for eating event–triggered EMAs. Time of day (afternoon odds ratio [OR] 0.60, 95% CI 0.42-0.85; evening OR 0.53, 95% CI 0.38-0.74) and whether other family members had also answered an EMA (OR 2.07, 95% CI 1.66-2.58) were significant predictors of compliance to time-triggered EMAs. Weekend status (OR 2.40, 95% CI 1.25-4.91) and deployment day (OR 0.92, 95% CI 0.86-0.97) were significant predictors of compliance to eating event–triggered EMAs. Participants confirmed that 76.5% (302/395) of the detected events were true eating events (ie, true positives), and the precision was 0.77. The proportion of correctly detected eating events did not significantly differ by participant age, gender, family role, or height (*P*>.05).

**Conclusions:**

This study demonstrates that EMA is a feasible tool to collect ground-truth eating activity and thus evaluate the performance of wearable sensors in the field. The combination of a wrist-worn smartwatch to automatically detect eating and a mobile device to capture ground-truth eating activity offers key advantages for the user and makes mobile health technologies more accessible to nonengineering behavioral researchers.

## Introduction

### Challenges to Dietary Assessment

A prevailing challenge in dietary and eating research is the ability to accurately measure dietary intake. Historically, the assessment of dietary intake and eating behaviors uses self-reporting tools [1,2], such as food diaries, food frequency questionnaires, and 24-hour dietary recalls [3,4]. All dietary assessment self-report methods have some level of measurement error (difference between measured and true values) [5,6]. Dietary data collected via self-report methods may be misreported because of biases, such as recall or memory bias (when a respondent erroneously recalls their dietary intake) and social desirability bias (when a respondent desires to present oneself positively) [7-9]. Studies have also found that those with certain characteristics (eg, obese weight status and body image dissatisfaction) are more likely to underreport their energy intake [10,11].

### Shifting Focus From Dietary Intake to Eating Behavior and Context

The field of nutritional epidemiology has produced an abundance of studies that have examined the role of *dietary intake* (ie, what and how much is consumed) in human health and disease—specifically, macronutrients (eg, fats and carbohydrates), types of food, quality of food, dietary patterns, and more [12]. Decades of laboratory-based and observational research indicate that dietary intake is a critical component of chronic disease prevention [13]. However, the measurement of diet in *free-living* populations remains a significant challenge in the field. In addition, even if public health researchers can easily and accurately track free-living dietary intake, dietary intake patterns are notoriously difficult to change long-term [14].

*Eating behaviors and patterns* (ie, food choices and motives and feeding practices) and *context* (who is eating, when, where, with whom, etc) also play a significant role in the development of obesity and other chronic diseases, including type 2 diabetes and heart disease [15-20]. These findings indicate that the patterns and features of eating events may be key contexts that shape dietary intake, and thus could be more malleable features of eating behavior that could be intervened. However, the field lacks appropriate behavioral theories that provide a richer understanding of how eating behaviors vary across contexts and across time [21,22].

### Technology-Assisted Dietary Assessment

Emerging technologies offer a potential solution for the accurate assessment of dietary intake by addressing the limitations of self-reported dietary assessment methods. The incorporation of technologies into dietary assessment can improve the quality and validity of dietary data by passively measuring eating in naturalistic settings over long periods with minimal user interaction [23]. Two emerging technological advances in dietary assessment tools include the following:

1. Ecological momentary assessment (EMA): a data collection technique in which one’s behavior is repeatedly sampled in real time and in context [24-26].
2. Wearable devices and sensors: allow for the passive collection of various data streams from the physical environment (eg, acoustic, visual, and inertial) [27].

EMA and wearable sensors are able to measure behavior near or just in time, thereby reducing or eliminating the recall bias that can affect retrospective self-report measures. In addition to improving the validity of data, these technologies offer the opportunity to measure eating behavior frequently and over long periods, allowing researchers to examine how it varies over multiple timescales (varies over the day, over the week, etc).

### Monitoring and Modeling Family Eating Dynamics Study

To address the limitations of traditional dietary assessment methods and theories, the Monitoring and Modeling Family Eating Dynamics (M2FED) study developed a sensor system that used smartphones as well as deployable and wearable sensors to collect synchronized real-time data on family eating behavior [28]. This study used the following: (1) wrist-worn smartwatches containing inertial sensors (accelerometer and gyroscope) to automatically detect arm movements and hand gestures associated with eating; (2) EMA via smartphone to confirm whether the eating occurred and to measure other contextual information, such as who was present during the eating event and the current mood of the respondent; and (3) Bluetooth proximity beacons to determine the approximate location of the smartwatches.

Rather than focusing on dietary intake (caloric intake, portion sizes, etc), this study took a novel approach by measuring eating behaviors (ie, food choices and motives and feeding practices) and context (who is eating, when, where, with whom, etc). Family eating dynamics have yet to be measured and modeled dynamically to better contextualize our understanding of social influence processes within family systems. This paper begins the first step toward producing new models that develop behavioral theory, and it may enable the identification of temporally specific processes and events within the family system that can be targeted for personalized, context-specific, real-time feedback.

### Assessing Validity of Wearable Sensors

The validity of using wearable sensors to automatically assess eating behavior and context has been tested in both laboratory and field settings [27,29-31], indicating that the performance of the wearable sensors decreases in naturalistic settings (compared with controlled laboratory settings). Studies have used a variety of sensors (eg, microphones, cameras, smartwatches, and electromyography electrodes) to measure various dietary outcomes, including bites, chewing, swallowing, and duration of eating occasions [27,29-33]. A review by Bell et al [27] indicates that there is still a strong reliance on retrospective self-report methods (eg, end-of-day food diaries) to determine ground-truth eating activity to evaluate wearable sensors in the field. Given the aforementioned limitations of retrospective self-report methods to accurately assess diet, the M2FED study used event-contingent EMA to determine ground-truth eating activity in families. The use of EMA offers unique methodological advantages, such as the following:

- The ability to measure behavior near or just in time, thereby reducing recall bias and reducing participant burden.
- The ability to measure behavior at the location in which it actually occurs, thereby maximizing ecological validity [24].

The validity of this method has been tested in a few in-field studies [34,35]; however, it has not yet been tested in a family-based study.

### Assessing Feasibility of EMA

One disadvantage of using technologies for data collection is the potential for participant noncompliance. A recent systematic review and meta-analysis by Wen et al [36] found that compliance rates among EMA studies in youth samples were suboptimal; the weighted average compliance rate was 78.3%, falling under the recommended 80% compliance rate [24]. Many studies have explored EMA compliance for various behaviors in various populations [36-40], but the compliance rate for a family-based EMA study is underexplored. A recent EMA study involving mothers and their children found that mothers’ presence may enhance children’s compliance with EMA questionnaires [41], suggesting that family members and other social relations may be leveraged to increase compliance in future EMA studies.

### Study Aims

Therefore, the overall purpose of this study is to report on participant compliance (feasibility) to the 2 distinct EMA protocols of the study (hourly time-triggered and eating event–triggered assessments) and on the performance (validity) of the wearable sensor in automatically detecting eating events in a family-based study. Specifically, the primary aims of this study include the following:

- Aim 1A—evaluate participant compliance with the EMA protocol, (1) overall, (2) for hourly time-triggered survey assessments, and (3) for eating event–triggered survey assessments—and aim 1B—evaluate the impact of time (time of day, day of week, and deployment day), age, gender, family role, and compliance of other family members (whether another participating family member j had answered a survey that had been received within 15 minutes of focal person i’s survey) on compliance.
- Aim 2A—evaluate the performance of the wrist-worn smartwatch to automatically detect eating events of participants at home—and aim 2B—determine whether there are systematic differences in the detection of eating events by age, gender, family role, and height.

## Methods

### Participants and Recruitment

#### Eligibility

The research team recruited families that contained at least two members (including at least one adult parent and one child between the ages of 11 and 18 years) living in Los Angeles County. Families with children aged <11 years were eligible to participate; however, children aged <11 years were not permitted to participate in the study. Families were not eligible to participate if one or more family members living at home did not primarily speak English. There were no demographic or disease-related exclusion criteria.

#### Method of Recruitment

Families were recruited in public spaces and at public events in Los Angeles County from May 2017 to August 2019. Snowball sampling was also used, such that participating families were offered an additional US $20 if they referred other eligible families that were successfully enrolled in the study.

All families that expressed interest and met the eligibility requirements were invited to participate in the study. An intake screening tool was administered over the phone by recruitment coordination staff to confirm eligibility before enrolling in the study.

This study was approved by the Institutional Review Board of the University of Southern California (UP-16-00227). All parents provided informed written consent, and all children provided assent.

### M2FED System

#### Overview

**Figure 1:**
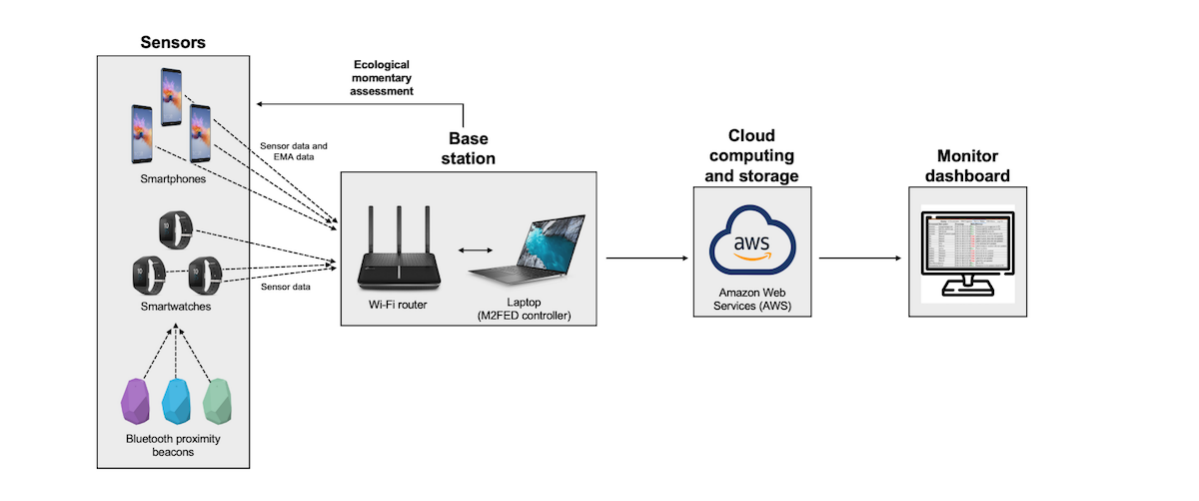
Overview of the Monitoring and Modeling Family Eating Dynamics cyberphysical system. EMA: ecological momentary assessment.

The primary objective of the M2FED study is to develop and deploy the M2FED cyberphysical system (Figure 1) in the homes of families. Cyberphysical systems can be defined as “physical and engineered systems whose operations are monitored, coordinated, controlled, and integrated by a computing and communication core” [42]. This novel system monitored *in-home* family eating behaviors in all participants. This system contained four primary components (1) sensors (including smartwatches, smartphones, and Bluetooth proximity beacons), (2) a base station, (3) an EMA subsystem, and (4) a remote monitoring subsystem, all of which were connected through a Wi-Fi router (Figure 1).

For the scope of this study, all data collected by the system were measured in the home (ie, no data were collected outside of the home).

#### Sensors

Participants were instructed to wear a Sony Smartwatch 3 (Android Wear operating system) on their dominant hand during all waking hours that they were in their home. The smartwatches were used to *automatically* detect eating-related hand-to-mouth (H-t-M) gestures for each participant at home and in real time. Arm movements and H-t-M gestures were detected via an algorithm that used motion data from the inertial sensors inside the smartwatch (accelerometer and gyroscope) [43]. If a cluster of at least two H-t-M gestures were detected within a 1-minute time frame, then the motion data were processed with a more sophisticated algorithm, and these *clusters* were then characterized as an *eating event*. An eating event can be defined as a set of H-t-M gestures, representing phenomena such as consuming a meal, snack, drink, or a combination of these consumption behaviors in which H-t-M gestures are clustered temporally. The technical details of the eating event detection algorithm are provided in detail elsewhere [43]. Participants were instructed to wear the smartwatch only at home and to not take it outside or wear it outside of the home. Consequently, data on H-t-M gestures and eating events that were determined by the proximity beacons that occurred outside of the home were discarded.

Participants were each provided with a Samsung Galaxy S7 smartphone (Android operating system) preprogramed with limited functioning. The smartphone app in which they responded to mobile questionnaires was pinned to the screen so that they could not access other apps on the smartphone. This smartphone was only intended for use as a data collection tool. Participants were instructed to keep their smartphones at home and not take it outside of the home. If a smartphone left home and was not within the range of the Wi-Fi router, the phone did not receive any mobile questionnaires. Consequently, data on participants’ states and behaviors outside of the home were not collected.

Estimote Bluetooth Low Energy proximity beacons were used to determine the approximate location of smartwatches of participants (including approximately which room the watches were in and whether they were still at home) during the study period. The beacons continuously broadcasted *packets* that included the unique media access control address of the Bluetooth interface, whereas the smartwatches periodically scanned for these *packets*. The smartwatches then recorded the received signal strength indicator (signal from the beacons), which indicated the proximity of the smartwatches to the beacons.

Typically, 1 to 2 beacons were placed on a wall in each living space at home (excluding bathrooms and bedrooms), and they required no further action by the participants during the study.

#### Base Station

A base station is a radio receiver and transmitter and a computing platform that serves as the hub of a local wireless network (the M2FED system). The base station for the M2FED system was a Lenovo ThinkPad laptop, which was placed in the home of the family for the duration of the study. The laptop was placed in a locked cage so that it could not be tampered with. The base station collected and processed the data received from the smartphones and smartwatches through the Wi-Fi router, and managed the EMA subsystem that ran on the laptop as well.

#### EMA Subsystem

EMA is a data collection technique in which one’s behavior is repeatedly sampled in a natural environment [24]. In this study, participants were assessed on several individual behaviors and states via mobile questionnaires sent to their smartphone approximately every hour during waking hours. Each smartphone had an app developed by the members of our research team installed on it. The app acted as a mobile questionnaire platform (ie, participants answered the questionnaires within the app interface).

The two types of EMAs that the participants received are as follows: (1) time-triggered mobile questionnaires and (2) eating event–triggered mobile questionnaires.

**Figure 2:**
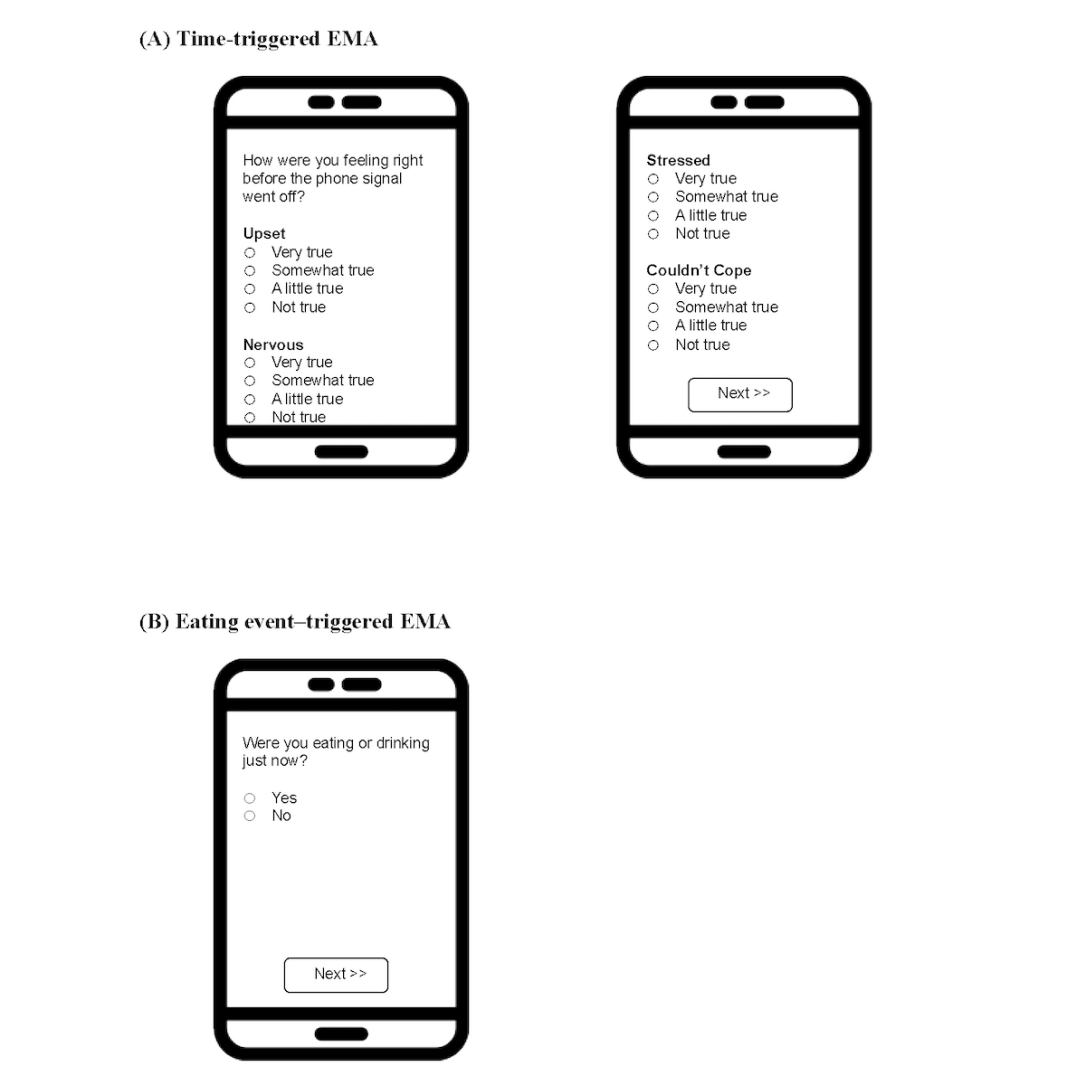
Examples of a time-triggered and eating event–triggered mobile questionnaire received on the phone of a participant. Figure 2A is an example of a time-triggered mobile questionnaire that the participants received on their phone during the study. It contains the first 4 questions of the questionnaire that measure negative affect. Figure 2B is an example of an eating event–triggered mobile questionnaire that the participants received on their phone during the study. It contains the first question of the questionnaire that measures whether the participant had just eaten or drank. EMA: ecological momentary assessment.

A *time-triggered* mobile questionnaire was sent to the participants’ smartphones every hour at the top of the hour (eg, 10 AM, 11 AM, 12 PM, etc; Figure 2A). The questionnaire included a brief validated positive affect and negative affect survey [44-47] (see Table 1 for the full list of questions).

Shortly after an eating event was detected for any given participant, an *eating event–triggered* mobile questionnaire was sent to the corresponding participant’s smartphone asking to confirm whether they had just eaten **(**Figure 2B**)**. If they confirmed that they had just eaten, then following this first question, they were asked a battery of survey items including previously validated measures of hunger and satiety [48], mindful eating [49], positive and negative affect [44-47], and with whom they were eating, if anyone (see Table 1 for the full list of questions). If the participant had not finished eating, they were given the option to request more time before filling out the questionnaire.

If they responded to the first question indicating that they had not just eaten, then they were asked to report what activity they had just completed. They were then asked to respond to validated measures of positive and negative affect [44-47].

**Figure 3:**
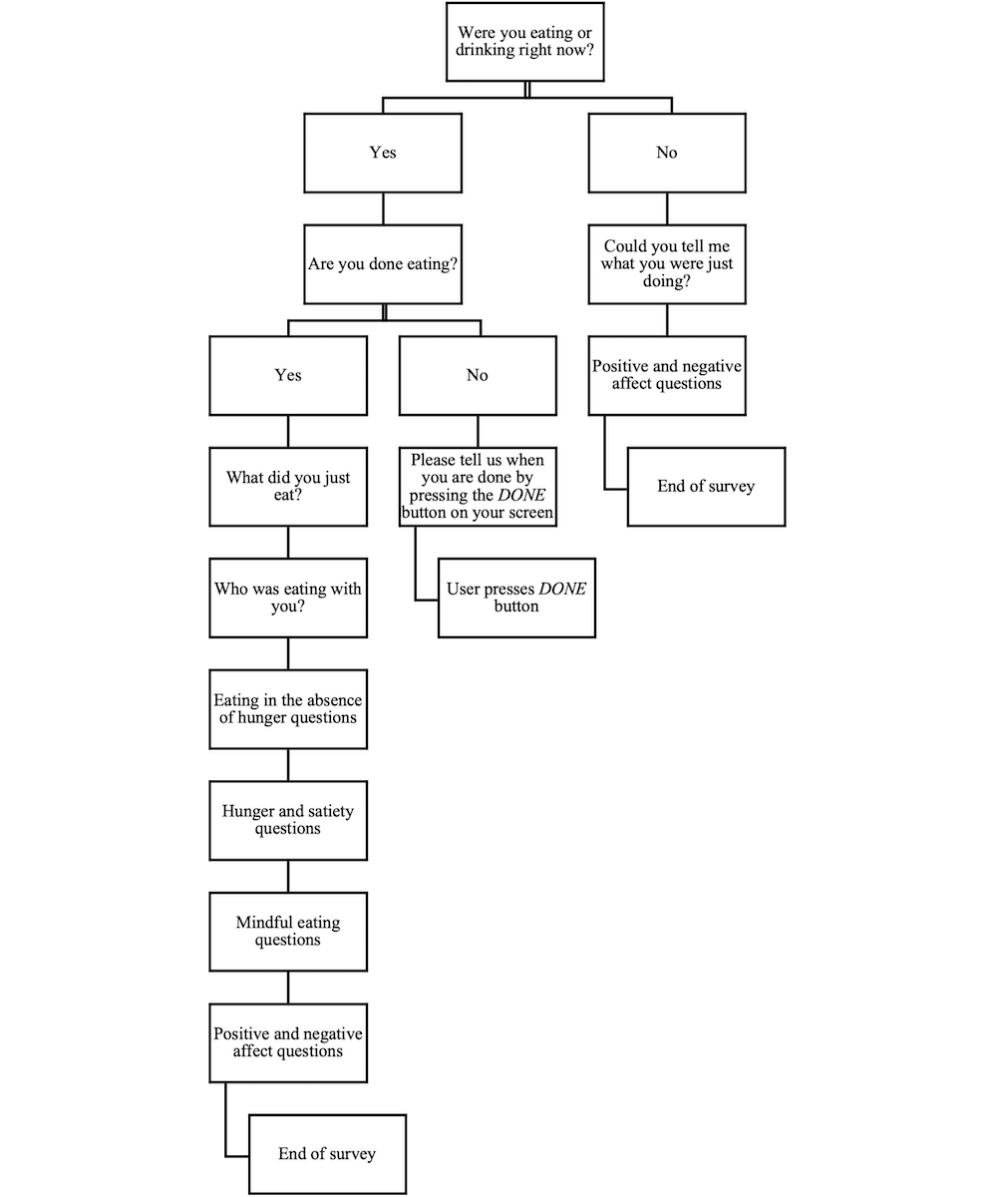
Eating event–triggered ecological momentary assessment question logic.

Figure 3 illustrates the full eating event–triggered EMA question logic. The full list of questions for the time-triggered and the eating event–triggered mobile questionnaires can be found in Table 1.

**Table 1:** Ecological momentary assessment (EMA) items.

Variable (subscale)			Items		Response options		Format
**Time-triggered EMA**							
	Positive and negative affect	How were you feeling right before the phone signal went off? (upset, nervous, stressed, could not cope, happy, great, cheerful, joyful)		Not at all A little Some Very		Separate screen for each of the 8 items	
**Eating event–triggered EMA**							
	Eating confirmation	Were you eating or drinking just now?		Yes No		—^a^	
	Eating type	What did you just eat?		Meal Snack Drink only		—	
	Social context	Who was eating with you? (check all that apply)		Nobody Spouse or partner Child(ren) Mother Father Sister(s) Brother(s) Grandparent Other family Friend(s) Other people		—	
	Eating in the absence of hunger—started eating	I *started* eating because (food looked, tasted, or smelled so good; others were eating; feeling sad or depressed; feeling bored; feeling angry or frustrated; feeling tired; feeling anxious or nervous; my family or parents wanted me to eat).		Not at all A little Some Very		Separate screen for each of the 8 items	
	Eating in the absence of hunger—kept eating	I *kept* eating because (food looked, tasted, or smelled so good; others were eating; feeling sad or depressed; feeling bored; feeling angry or frustrated; feeling tired; feeling anxious or nervous; I wanted to finish the food on my plate).		Not at all A little Some Very		Separate screen for each of the 8 items	
	Hunger level before eating	How hungry were you right before you ate?		0=Not at all hungry 100=Greatest imaginable hunger		Sliding scale 0 to 100	
	Satiation level after eating	How full were you right after you ate?		0=Not at all full 100=Greatest imaginable fullness		Sliding scale 0 to 100	
	Mindful eating	Before the beep, while I was eating (My thoughts were wandering while I ate; I was thinking about things I need to do while I ate; I ate so quickly that I did not taste anything).		Very true Somewhat true A little true Not true		Separate screen for each of the 3 items	
	Positive and negative affect	How were you feeling right before the phone signal went off? (upset, nervous, stressed, could not cope, happy, great, cheerful, joyful)		Not at all A little Some Very		Separate screen for each of the 8 items	

^a^No additional formatting notes.

#### Participation Windows

Before a family’s deployment started, all participants were individually asked about the time at which they normally woke up and the time at which they normally went to bed. The participants were limited to only 1 *personalized participation window* for the study. Therefore, they could not have different windows for Monday versus Tuesday and weekday versus weekend. If the times at which they woke up or went to bed varied extensively among days, then they were asked to provide a time frame that generally worked for all days. The purpose was to create *personalized participation windows* to account for variations in the daily routines and sleeping patterns of the participants. For the duration of the study, the participants only received EMAs during their personalized participation window. For example, if the window of a participant was from 6:30 AM to 11:00 PM, then they only received EMAs during that period.

#### Remote Monitoring Subsystem

The monitoring subsystem was used to monitor the status of the M2FED system in real time [50]. The subsystem monitored several things, including the battery status and network connection of the smartwatches, smartphones, and base station; the processes running on the base station; the detected eating events; and whether participants responded to any given EMA sent to their smartphones. When the monitoring system detected an issue (eg, the base station was no longer connected to the router), an email was sent to the research team to alert them of the issue.

### Procedures

Following enrollment, 2 members of the research team visited the home of each recruited family 2 separate times.

#### Visit 1

During the first home visit, the team went to the participants’ home to obtain consent from all participating family members, take body measurements of the participants using a research-grade Tanita scale (Model TBF 300) and stadiometer, administer baseline surveys, and install the components of the cyberphysical system around the home (all *living spaces*, not including bedrooms or bathrooms).

The base station, Wi-Fi router, and Bluetooth beacons were placed in a discrete location in the home of the family, so they could run without interference for the duration of the study. Samsung smartphones and Sony smartwatches were provided to all participating family members for the duration of the study (all features except answering questionnaires were turned off). Each phone and watch was designated to a specific participant and labeled with their name so that they knew which devices were their own. The team instructed the family on how to properly wear, charge, and care for the smartwatches and how to answer an EMA on the smartphones. The family was instructed to wear the watch at all times when they were at home and to answer all EMA questionnaires they received when they were at home. They were also instructed to leave their designed phone and watch at home when they left home to prevent the devices from getting damaged or lost while outside of the home.

Upon leaving the visit, family members underwent approximately 14 consecutive days of (1) use of a smartphone to complete hourly time-triggered and eating event–triggered mobile questionnaires, up to once every hour during waking hours; and (2) eating event monitoring, in the form of a wrist-worn smartwatch during waking hours.

#### Visit 2

At the final home visit, approximately 2 weeks following the first home visit, the research team terminated data collection, and all equipment was uninstalled and removed from the homes. Each participant received US $100 in a Visa gift card format as compensation for the 2-week study.

### Measures

#### Eating Events

During the 2-week assessment period, participants were asked to wear their dedicated smartwatch on their dominant wrist at all times while they were home during waking hours. Automatic eating event detection software on the smartwatches developed by our research team [43] collected the timestamps (approximate start and end times in the format mm/dd/yyyy, hh:mm:ss) for all detected eating events that occurred while the watch was worn. After an eating event was detected, participants received a brief mobile questionnaire on their study phones to confirm whether the detected eating event was a true event. The first question on the questionnaire was “Were you eating or drinking just now?” If the participant responded “No,” they were asked to report what they were doing. Options included *using my phone, smoking, fixing my hair, putting on sunscreen or lotion, or other* with an open text field. If the participant responded “Yes,” they were asked to report on a range of momentary measures, such as hunger level before the eating event and with whom they were eating. The full list of questions for the time-triggered and the eating event–triggered mobile questionnaires can be found in Table 1.

#### EMA Questionnaires

Timestamps (format: mm/dd/yyyy, hh:mm:ss) when the hourly time-triggered and eating event–triggered mobile questionnaires were sent to and received by the smartphones of participants were obtained from the monitoring system. In addition, the responses of the participants to the questionnaires were obtained from the monitoring system.

#### Timing

*Time of day* at which and *day of week* on which an eating event occurred was calculated using the timestamp of the detected eating events. The time of day at which the eating event occurred was stored in hh:mm:ss format. The *lubridate* R package [51] was used to convert the date on which the eating event occurred (format: mm/dd/yyyy) to the day of corresponding week (Monday, Tuesday, etc), which was then converted to weekday (Monday, Tuesday, etc) and weekend (Saturday or Sunday).

#### Anthropometrics

During home visit 1, height (cm), weight (kg), and body fat percentage (%) were measured in all participants in a private section of the home, using a portable stadiometer and a research-grade Tanita scale (model TBF 300).

#### Demographics

During home visit 1, participants were asked to provide basic demographic information via a paper-based questionnaire, including their current age (years), gender (female or male), race (Hispanic or Latino, Asian or Pacific Islander, White, Black or African American, American Indian or Native American, Mixed, or other), Hispanic or Latino ethnicity (Yes, No, or Do not know), and family role (mother, father, child, grandparent, aunt, uncle, and others).

### Analytic Approach

#### Data Processing

**Figure 4:**
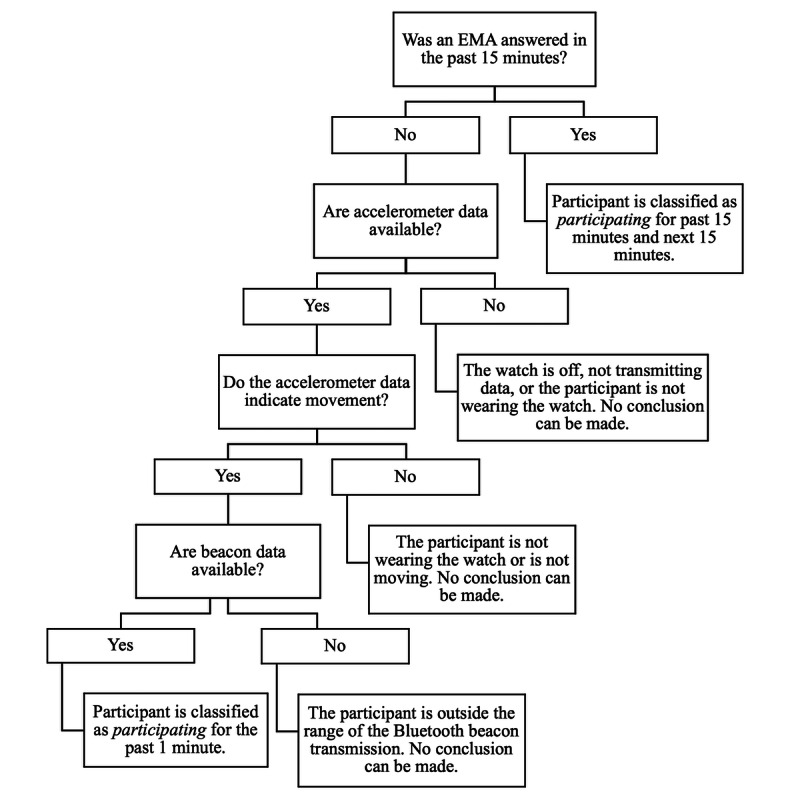
Decision tree to determine when study participants were participating at home. EMA: ecological momentary assessment.

A limitation of the EMA sampling protocol of the M2FED study was that the study phones of participants (which were instructed to be kept at home at all times) received hourly, time-triggered surveys regardless of whether the participants themselves were at home or not (at school or work, running errands, etc). This means that the time frame in which any given participant was at home and participating in the study was not necessarily continuous. Although we do not possess the ground truth for presence of the participants at home (eg, no cameras and no self-report diaries), our research team generated a *participation algorithm* using the EMA system, proximity sensors, and accelerometer in the watch to identify time intervals in which we were confident that the participants were both at home and actively participating in the study (ie, answering EMAs or wearing the smartwatch; Figure 4).

**Figure 5:**
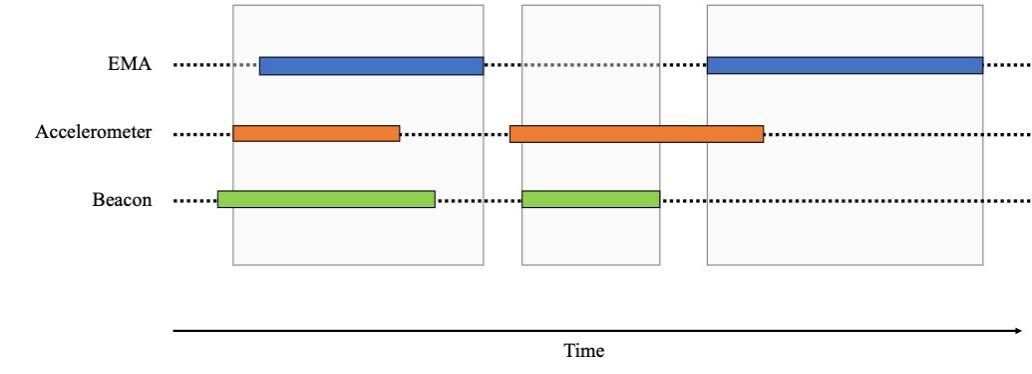
Example of participation time intervals for a participant. In this example, the shaded gray regions indicate the valid participation time intervals for this participant. In the first interval, we see that the participant answered an ecological momentary assessment (EMA), and there were available data from the accelerometer and beacon. In the second interval, the participant did not answer an EMA, but there were available data from the accelerometer and beacon. In the third interval, the participant answered an EMA and there were some available data from the accelerometer.

If participants had answered an EMA at time t, then we assigned their status as *participating* for the 30-minute interval surrounding time t (ie, from t −15 to t +15 minutes). For times outside the EMA interaction windows, we used data from the sensors (smartwatch accelerometer and Bluetooth beacons) to determine the status of the participants. For every minute, if the accelerometer data of the smartwatch was both available (ie, not missing for that minute) and indicated movement (ie, the frequency and instantaneous changes of the sensor signal was above a threshold, representing change in the signal because of movement) and beacon data were available, then they were classified as *participating* for that 1-minute interval. Contiguous minute intervals with *participating* status were merged to acquire larger time intervals. For each participant, these *participation* time intervals were calculated, and the union of all intervals (Figure 5) was used as the valid time interval in the analyses.

#### Data Analyses

#### Individual- and Family-Level Characteristics

The mean and SD or the count and proportion of the analytic sample's age, BMI, gender, race, and ethnicity were calculated and reported by family role (child or parent). At the family level, the count and proportion of the type of household of the family (1- or 2-parent household), number of children living at home, and average length of family deployment were reported.

#### EMA Characteristics

The mean and SD of EMAs received per family, received per person, and received per person per day were calculated after applying the participation algorithm to the EMA data. The frequency distribution of EMAs by family role and time of day was calculated.

#### Primary Analyses

To test study aim 1A, EMA compliance was calculated as follows (*i* can be values from 1 to *n*, where *n* represents the number of participants in the study):

Overall compliance to EMAs for participant_*i*_ = total number of EMAs answered by participant_*i*_ / total number of EMAs received at home by participant_*i*_  **(1)**

Compliance to *time-triggered* EMAs for participant_*i*_ = total number of time-triggered EMAs answered / total number of time-triggered EMAs received at home by participant_*i*_  **(2)**

Compliance to *eating event–triggered* EMAs for participant_*i*_ = total number of eating event–triggered EMAs answered / total number of eating event–triggered EMAs received at home by participant_*i*_  **(3)**

Means and SDs of overall compliance to EMAs, compliance to time-triggered EMAs, and compliance to eating event–triggered EMAs were also calculated across all participants.

To test study aim 1B, the unit of analysis was every EMA that was sent to and received by the smartphones of the participants throughout the span of the 2-week data collection period. Compliance (dependent variable) was calculated as *1* if the questionnaire was answered and as *0* if the survey was not answered. A logistic regression model was fitted with the following independent variables: type of EMA (time-triggered and eating event–triggered), time of day (morning, defined as midnight to 11:59:59 AM; afternoon, defined as noon to 16:59:59 PM; and evening, defined as 17:00:00 PM to 23:59:59 PM), day of week (weekday, defined as Monday through Friday; and weekend, defined as Saturday and Sunday), gender (male or female), family role (parent, child, or other), and social factors (whether another participating family member *j* had answered a survey that had been received within 15 minutes of the focal person *i*’s questionnaire).

To test study aim 2A, we evaluated the performance of the smartwatch by computing the following metrics for all eating events automatically detected during deployments:

True positives = cases in which an eating event actually *occurred*, and that eating event was *correctly* detected by the smartwatch algorithm

False positives = cases in which an eating event actually *did not* occur, but an eating event was *erroneously* detected by the smartwatch algorithm.

Precision = true positives / (true positives + false positives)  **(4)**

To test study aim 2B, nonparametric methods were used to determine whether there were differences in the detection of eating events by participant age, gender, family role, and height. The metric we used to compare across demographic groups was the following:

Proportion of correctly detected eating events for participant_*i*_ = true positives for participant_*i*_ / total number of detected eating events for participant_*i*_  **(5)**

If any participant had received fewer than 3 eating event–triggered EMAs, their data were excluded from this analysis.

For categorical variables with 2 groups (ie, gender), the appropriate assumptions were tested, and then the Mann–Whitney *U* test was used to test for equality of central tendency of the 2 distributions; for categorical variables with 3 or more categories (ie, family role), the Kruskal-Wallis test was used. Finally, for continuous variables (ie, height [cm] and age [years]), the appropriate assumptions were tested, and Spearman rank correlation was used to measure the strength and direction of the relationship between the continuous variable and the proportion of correctly detected events.

#### Missing Data

There were no missing anthropometric or demographic data. Similarly, there were no missing data on detected eating events and corresponding variables, including time of eating event and day of eating event; however, there were missing data for time-triggered and eating event–triggered EMAs.

##### Missingness Attributed to Technical Issues

Preliminary analyses indicated that not all EMAs that were *sent* to the study phones of the participants by the M2FED system were *received* by the phone. The M2FED system ran independently on the base station regardless of the network connection, and therefore *sent* EMAs regardless of network connection. However, a network connection was needed for the phone to successfully *receive* the EMA.

Although we do not have data that explain why this happened at every instance, we know from in-the-field troubleshooting and from accounts given by participants that at least a portion of the nonreceived EMAs resulted from (1) network connection issues at home (ie, the router was not working and the EMAs could not be received on the phone) and (2) EMA app failure (ie, the EMA app on the phone failed to work properly).

For these analyses, we removed any EMAs that were sent by the system but were not received by the phone.

##### Missingness Attributed to Participant Nonresponse or Partial Response

The different types of missing data that we encountered were because of participant nonresponse (ie, participants did not respond to any EMA questions) or partial responses (ie, participants did not respond to all EMA questions).

For aim 1 analyses, if participants did not respond to *any* questions on a given mobile questionnaire, then this EMA was labeled as *received but not answered*. If participants did not respond to *all* questions, then this EMA was labeled as *received and partially answered*. These EMA observations were kept in the data set to calculate EMA compliance.

For aim 2 analyses, if participants did not respond to at least the *first* question on a given eating event–triggered EMA (“Were you eating or drinking just now?”), then this EMA observation was removed from the data set.

*Statistical software* R (version 4.0.2) was used to perform these analyses.

## Results

### Individual- and Family-Level Characteristics

**Figure 6:**
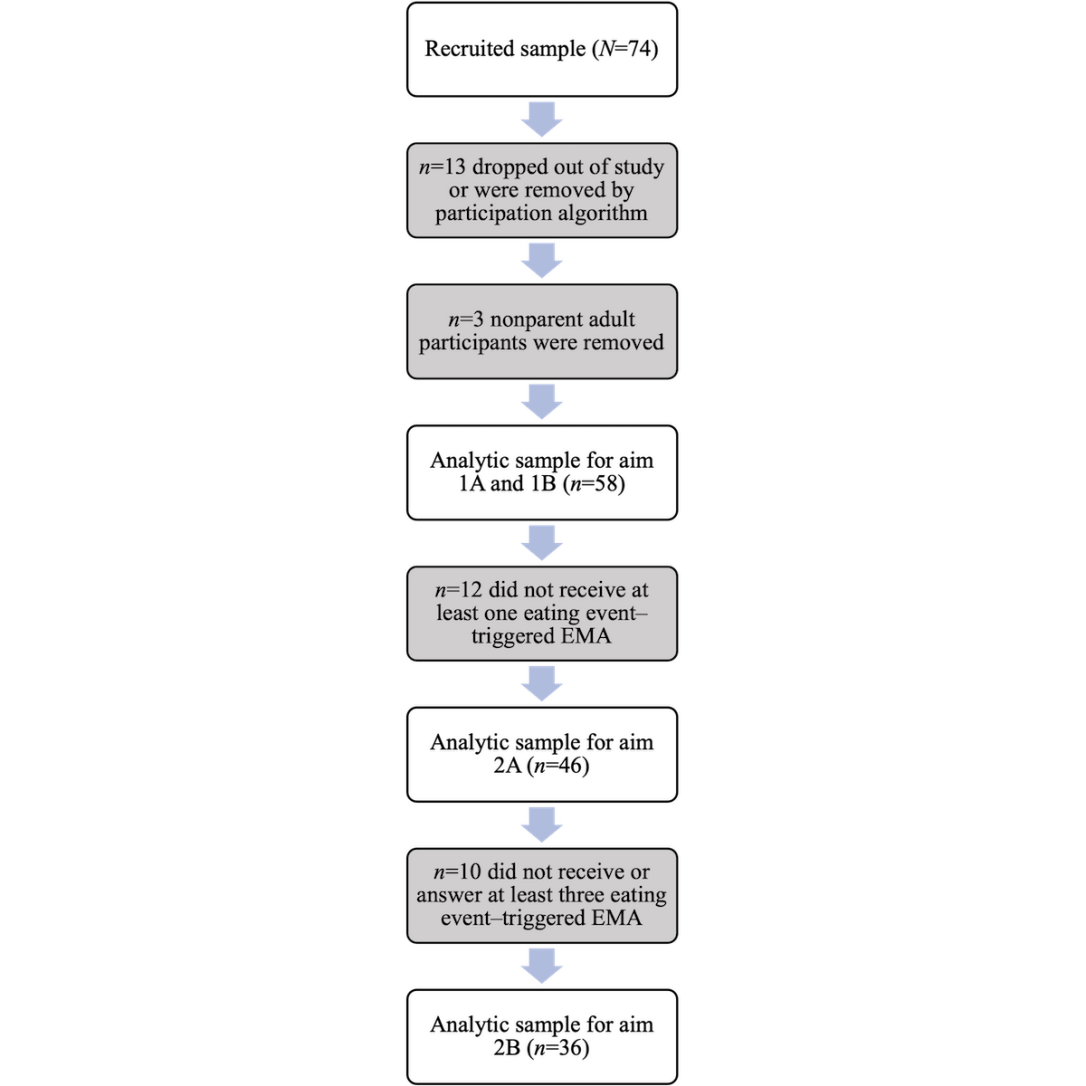
Flow of participants in the Monitoring and Modeling Family Eating Dynamics study. Participants may not have received an eating event–triggered ecological momentary assessment (EMA) as no eating event was detected by the system or technical issues prevented the EMA from sending.

A total of 74 participants from 20 families were enrolled in the M2FED study. In all, 18% (13/74) of participants dropped out of the study or were removed from the data set if their participation (as determined by the participation algorithm) was 0% (ie, they did not answer any EMAs and never wore the smartwatch; Figure 6).

In addition, the data from 4% (3/74) nonparent adult participants made up approximately 1.44% (61/4232) of the EMAs received, so these participants were removed from the analytic sample as well. The remaining 78% (58/74) of participants included in the analytic sample did not significantly differ from the enrolled sample (N=74) by age, gender, or parent role (*P*>.05; Table 2).

Of the 58 participants, 43% (n=25) were parents and 57% (n=33) were children. On average, children were aged 15.12 years (SD 3.97 years) and parents were aged 43.72 years (SD 6.71 years). There were 39% (13/33) female children and 68% (17/25) female parents. In all, 61% (20/33) of children and 68% (16/25) of parents identified as Hispanic or Latino (Table 3).

Of the 20 enrolled families, most (17/20, 85%) were 2-parent households, 15% (3/20) of the families had 1 child living at home, 75% (15/20) of the families had 2 children, 5% (1/20) of the families had 3 children, and 5% (1/20) of the families had 4 children (Table 4). On average, family deployments lasted 14.90 days (SD 3.13 days).

**Table 2:** Comparison of recruited sample and analytic samples.

Characteristics	Values						
	Recruited sample (N=74)	Analytic sample for aim 1A and 1B (n=58)	*P* value^a^	Analytic sample for aim 2A (n=46)	*P* value^a^	Analytic sample for aim 2B (n=36)	*P* value^a^
Age (years), mean (SD)	28.91 (15.79)	27.45 (15.23)	.59	28.76 (15.51)	.96	26.67 (14.83)	.47
Sex (female), n (%)	37 (50)	30 (52)	.78	24 (52)	.81	20 (56)	.49
Parent (yes), n (%)	32 (43)	25 (43)	.99	21 (46)	.77	15 (42)	.97

^a^*P* values were calculated by comparing the analytic sample to the recruited sample. Welch 2 independent sample 2-tailed *t* test was used for continuous variables (ie, age), and Pearson chi-square test was used for categorical variables (ie, sex and parent).

**Table 3:** Individual-level characteristics of the Monitoring and Modeling Family Eating Dynamics analytic sample (N=58), by family member role.

Characteristics			Child (n=33)^a^		Parent (n=25)^a^
Age (years), mean (SD)			15.12 (3.97)		43.72 (6.71)
Sex (female), n (%)			13 (39)		17 (68)
**Race and ethnicity, n (%)**					
	Asian or Pacific Islander	1 (3)		1 (4)	
	Black or African American	2 (6)		1 (4)	
	Hispanic or Latino	20 (61)		16 (68)	
	White	4 (12)		4 (16)	
	Mixed	6 (18)		1 (4)	
	Other	0 (0)		1 (4)	
BMI^b^ percentile (n=53), mean (SD)			22.36 (4.66)		32.90 (7.38)

^a^The percentages presented are column percentages.

^b^BMI: body mass index.

**Table 4:** Family-level and deployment-level characteristics of the Monitoring and Modeling Family Eating Dynamics study families (N=20).

Characteristics			Values
**Number of parents living at home, n (%)^a^**			
	1-parent household	3 (15)	
	2-parent household	17 (85)	
**Number of children living in the home, n (%)^a^**			
	1 child	3 (15)	
	2 children	15 (75)	
	3 children	1 (5)	
	4 children	1 (5)	
Deployment length (days), mean (SD)			14.90 (3.13)

^a^The percentages presented are column percentages.

### EMA Characteristics

In total, 15,010 EMAs (14,348/15,010, 95.59% time-triggered and 662/15,010, 4.41% eating event–triggered) were sent by the M2FED system and received by study phones of the participants. After filtering the data through the participation algorithm, 27.78% (4171/15,010) EMAs remained in the data set: 88.95% (3710/4171) of which were time-triggered and 11.05% (461/4171) were eating event–triggered (Table 5).

**Figure 7:**
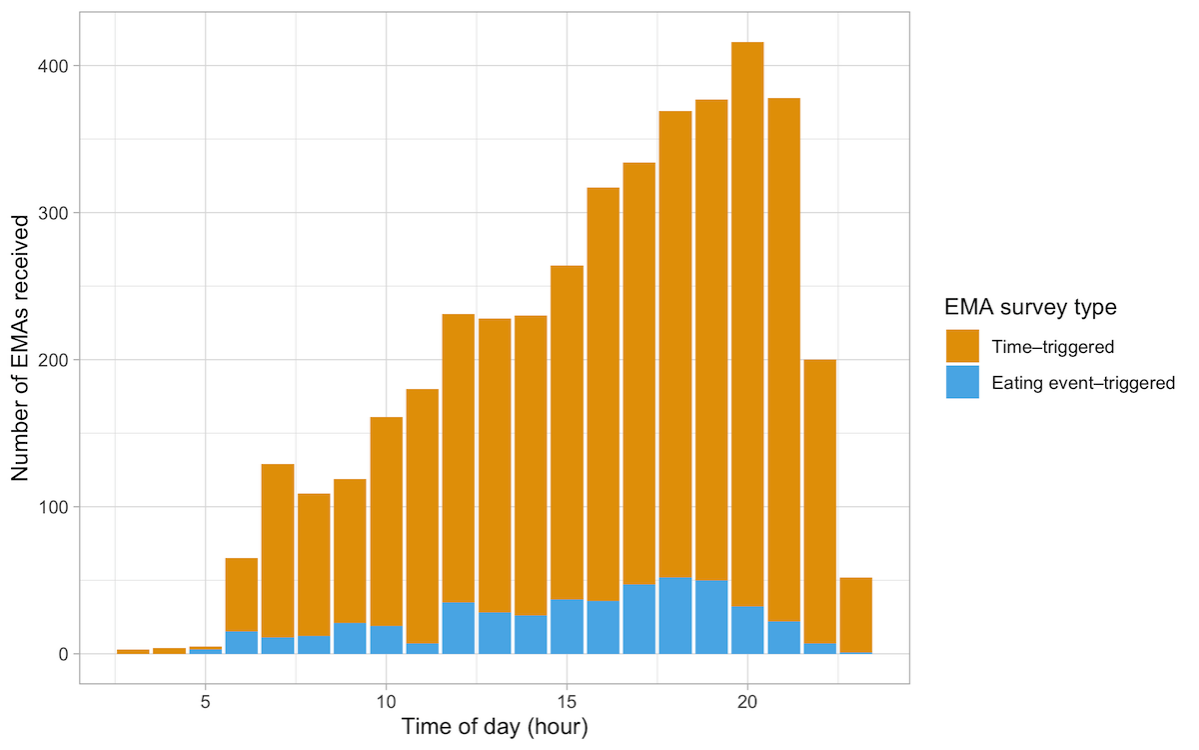
Distribution of ecological momentary assessments (EMAs) received across the time of day (hour), by EMA survey type.

On average, families received 209.0 EMAs (SD 89.4; range 86-391), and individuals received 71.9 EMAs (SD 34.3; range 8-176) each. Participants received, on average, 64.0 time-triggered EMAs (SD 31.3; range 8-147) and 8.0 eating event–triggered EMAs (SD 8.9; range 0-40) across the deployment. The daily average number of EMAs received per person was 5.2 (SD 2.7; range 0.6-11.7) for all EMAs, 4.7 (SD 2.4; range 0.3-10.2) for time-triggered EMAs, and 0.6 (SD 0.6; range 0-2.7) for eating event–triggered EMAs (Table 5). Of the 4171 total EMAs, 18.58% (775/4171) were received in the morning, 30.46% (1270/4171) in the afternoon, and 50.97% (2126/4171) in the evening. Of the 461 eating event–triggered EMAs, most, 45.8% (211/461), were sent in the evening (Figure 7). Children received 57.52% (2399/4171), fathers received 10.72% (447/4171), and mothers received 31.77% (1325/4171) of the total EMAs. Of the 461 eating event–triggered EMAs, 49.9% (n=230) were received by children, 7.4% (n=34) by fathers, and 42.7% (n=197) by mothers.

**Table 5:** Ecological momentary assessment (EMA) summary statistics after applying participation algorithm, by prompt type.

Type of EMA	Total EMAs received, N	EMAs received per family, mean (SD; range)	EMAs received per person, mean (SD; range)	EMAs received per person per day, mean (SD; range)
All EMA	4171	209.0 (89.4; 86-391)	71.9 (34.3; 8-176)	5.2 (2.7; 0.6-11.7)
Time-triggered EMA	3710	186.0 (84.3; 77-356)	64.0 (31.3; 8-147)	4.7 (2.4; 0.3-10.2)
Eating event–triggered EMA	461	23.0 (17.2; 3-69)	8.0 (8.9; 0-40)	0.6 (0.6; 0-2.7)

### Participant Compliance

**Figure 8:**
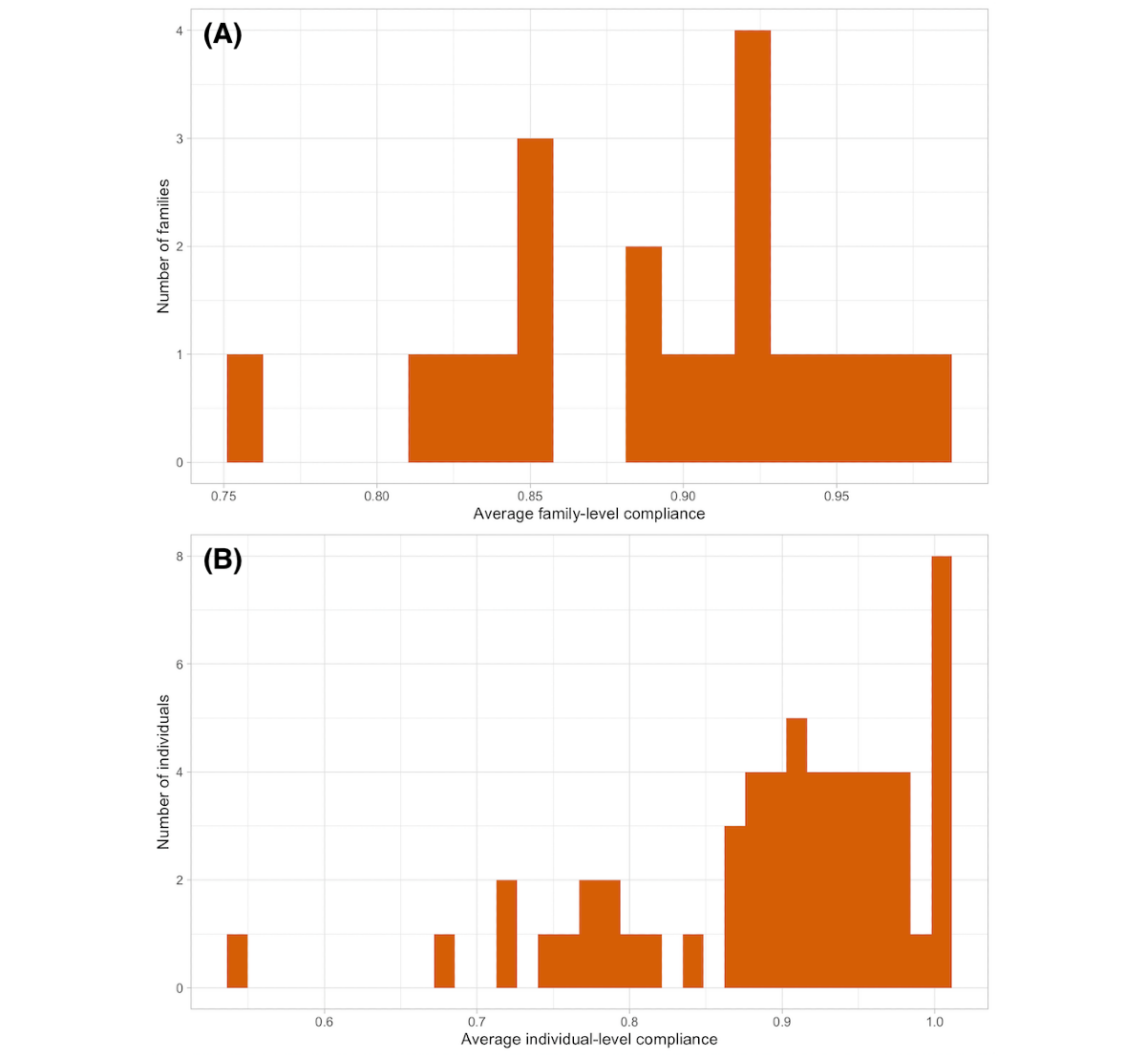
Distribution of (A) family-level and (B) individual-level compliance.

The overall compliance rate across the 20 deployments was 89.26% (3723/4171) for all EMAs, 89.7% (3328/3710) for time-triggered EMAs, and 85.7% (395/461) for eating event–triggered EMAs (Table 6). The average family-level compliance was 89.4% (SD 5.74%; range 75.7%-98.1%) for all EMAs, 89.8% (SD 5.84%; range 75.8%-98.7%) for time-triggered EMAs, and 85.9% (SD 14.3%; range 55.6%-100%) for eating event–triggered EMAs. At the individual-level, the average compliance for all EMAs was 89.6% (SD 9.5%; range 53.8%-100%), for time-triggered EMAs was 89.5% (SD 10.1%; range 50%-100%), and for eating event–triggered EMAs was 88% (SD 17.5%; range 28.6%-100%). The distributions of individual- (Figure 8A) and family-level compliance (Figure 8B) are shown in Figure 8.

**Table 6:** Ecological momentary assessment (EMA) compliance rates after applying participation algorithm, by prompt type.

Type of EMA	Total EMAs received, N	Total EMAs answered (compliance), n (%)	Family-level compliance (%), mean (SD; range)	Individual-level compliance (%), mean (SD; range)
All EMA	4171	3723 (89.3)	89.4 (5.74; 75.7-98.1)	89.6 (9.5; 53.8-100)
Time-triggered EMA	3710	3328 (89.7)	89.8 (5.8; 75.8-98.7)	89.5 (10.1; 50-100)
Eating event–triggered EMA	461	395 (85.7)	85.9 (14.3; 55.6-100)	88.0 (17.5; 28.6-100)

### Predictors of Compliance

Three separate logistic regression models were fitted with the following data sets: (1) all EMAs, (2) time-triggered EMAs, and (3) eating event–triggered EMAs.

Results from the first model indicate that time of day and whether other family members had also answered an EMA were significant predictors of compliance to all EMAs (Table 7). Participants were 37% less likely (odds ratio [OR] 0.63, 95% CI 0.46-0.86) to respond to an EMA in the afternoon and 39% less likely (OR 0.61, 95% CI 0.45-0.81) to respond to an EMA in the evening compared with the morning (reference group). Participants were 91% more likely (OR 1.91, CI 1.56-2.34) to respond to an EMA if another family member had responded to an EMA in the surrounding 30-minute time interval.

The results from the second model indicate that time of day and whether other family members had also answered an EMA were significant predictors of compliance to time-triggered EMAs (Table 7). Participants were 40% less likely (OR 0.60, 95% CI 0.42-0.85) to respond to a time-triggered EMA in the afternoon and 47% less likely (OR 0.53, 95% CI 0.38-0.74) to respond to a time-triggered EMA in the evening than in the morning (reference group). Participants were approximately 2 times as likely (OR 2.07, 95% CI 1.66-2.58) to respond to a time-triggered EMA if another family member had responded to any EMA in the surrounding 30-minute time interval.

Results from the third model indicate that weekend status and deployment day were significant predictors of compliance to eating event–triggered EMAs (Table 7). Participants were 2.4 times as likely (OR 2.40, 95% CI 1.25-4.91) to respond to an eating event–triggered EMA on the weekend, than on a weekday. Participants were 8% less likely (OR 0.92, 95% CI 0.86-0.97) to respond to an eating event–triggered EMA for every 1-day increase in deployment day.

**Table 7:** Logistic regression model results, examining predictors of compliance^a^.

Characteristics	Model 1: all EMAs^b^		Model 2: time-triggered EMAs		Model 3: eating event–triggered EMAs	
	*β* (SE)	OR^c^ (95% CI)	*β* (SE)	OR (95% CI)	*β* (SE)	OR (95% CI)
Intercept	*2.17*^d,e^ (*0.27*)	8.75 (5.20-14.82)	*2.22*^e^ (*0.29*)	9.22 (5.24-16.36)	*2.41*^f^ (*0.74*)	11.15 (2.65-48.64)
Age (years)	.00 (0.01)	1.00 (0.98-1.03)	.00 (0.01)	1.00 (0.98-1.02)	.02 (0.03)	1.02 (0.96-1.08)
Afternoon	−*.47*^f^ (*0.16*)	0.63 (0.46-0.86)	−*.51*^f^ (*0.18*)	0.60 (0.42-0.85)	−.35 (0.38)	0.71 (0.33-1.46)
Evening	−*.50*^f^ (*0.15*)	0.61 (0.45-0.81)	−*.63*^e^ (*0.17*)	0.53 (0.38-0.74)	.28 (0.38)	1.32 (0.62-2.75)
Weekend, yes	.06 (0.11)	1.06 (0.86-1.31)	−.06 (0.12)	0.95 (0.75-1.19)	*.87*^g^ (*0.35*)	2.40 (1.25-4.91)
Deployment day	−.02 (0.01)	0.98 (0.96-1.01)	−.01 (0.01)	0.99 (0.97-1.01)	−*.09*^f^ (*0.03*)	0.92 (0.86-0.97)
Female, yes	.19 (0.15)	1.21 (0.90-1.65)	.31 (0.17)	1.37 (0.98-1.92)	−.65 (0.43)	0.52 (0.22-1.22)
Mother	−.01 (0.34)	0.99 (0.51-1.93)	.06 (0.36)	1.06 (0.53-2.16)	−.65 (1.07)	0.52 (0.06-4.56)
Father	−.42 (0.35)	0.66 (0.33-1.30)	−.37 (0.38)	0.69 (0.33-1.47)	−.64 (0.93)	0.53 (0.08-3.26)
Others answered, yes	*.65*^e^ (*0.10*)	1.91 (1.56-2.34)	*.73*^e^ (*0.11*)	2.07 (1.66-2.58)	−.02 (0.30)	0.99 (0.54-1.76)

^a^Akaike information criteria is 2805.16, 2417.32, and 375.57 for models 1-3, respectively. Bayesian information criteria is 2868.52, 2479.50, and 416.91 for models 1-3, respectively.

^b^EMA: ecological momentary assessment.

^c^OR: odds ratio.

^d^Values indicate significant estimates.

^e^*P*<.001.

^f^*P*<.01.

^g^*P*<.05.

### Smartwatch Algorithm Evaluation

At least one eating event was automatically detected during the deployment for 46 participants. This subsample (ie, the analytic sample for aim 2A) did not significantly differ from the enrolled sample (N=74) by age, gender, or parent role (*P*>.05; Table 2).

A total of 461 eating events were automatically detected using the smartwatch algorithm across these 46 participants. Participants responded to 85.7% (395/461) of the corresponding eating event–triggered EMAs. Participants confirmed that 76.5% (302/395) of the detected events were true eating events (ie, true positives) and 23.5% (93/395) were not true eating events (ie, false positives). For approximately one-third of these false positives, participants reported that they were using their phones at the time. The calculated precision measure, that is, the number of true positives divided by the sum of true positives and false negatives, was 0.77.

### Differences in Eating Event Detection

At least three eating event–triggered EMAs were received by 36 participants. This subsample (ie, the analytic sample for aim 2B) did not significantly differ from the enrolled sample (N=74) by age, gender, or parent role (*P*>.05; Table 2). For this subsample, the average individual-level proportion of correctly detected eating events (true positives / total number of detected eating events) was 78.5% (SD 19%; range 30%-100%). In all, 72% (26/36) of the analytic sample had at least one falsely detected eating event (false positive).

Neither age (years) nor height (inches) was significantly correlated with the proportion of correctly detected eating events (r_s_=0.24, *P*=.17 and r_s_=−0.12, *P*=.52, respectively). The average individual-level proportion of correctly detected eating events for women was 82.1% (SD 20.4%; range 30%-100%) and was 74% (SD 16.6%; range 50%-100%) for men. The difference between the 2 groups was not significant (W=112; *P*=.13). The average individual-level proportion of correctly detected eating events for children was 74.3% (SD 19.3%; range 30%-100%), for fathers was 76.1% (SD 21.5%; range 58.3%-100%), and for mothers was 86.5% (SD 16.8%; range 54.5%-100%). The differences among these 3 groups were not significant (Kruskal–Wallis *χ*^2^_2_=2.998; *P*=.22).

## Discussion

The M2FED study sought a dramatically different mobile health (mHealth) approach to obesity prevention and intervention by not focusing directly on diet and activity, but rather on family eating dynamics. An in-home sensor system was developed and deployed to monitor family eating dynamics in real time and context.

### Evaluating EMA Compliance

After applying our customized participation algorithm, we found that both individual- and family-level compliance rates to the EMA protocols of the study were relatively high (both greater than the recommended 80%) [24]. Compliance was significantly higher in the mornings overall and higher on the weekends for eating event–triggered EMAs, which supported the informal feedback we received from participants that they were more likely to *participate* (ie, respond to EMAs and wear the smartwatch) when they did not need to go to work or school (typically the weekend days). We also saw that overall compliance decreased as the 2-week study went on, most likely attributable to participant fatigue.

One particularly interesting finding was that participants were significantly more likely to answer an EMA if another family member had answered an EMA in a similar time frame. A similar finding was reported by Dzubur et al [41], in which mother-child dyads were more likely to comply with prompts when they were together. Although the overarching aims of the M2FED study were to measure the social influence of family members on eating behavior, this finding also indicates that social influence came into play in other parts of the study as well. Drawing from the social psychology field, several social mechanisms could partially explain these findings. For instance, an expectation could have been set early on in particular families to answer the EMA prompts, thus establishing a social norm for EMA compliance [52,53]. Similarly, some individuals may have been inclined to answer EMA prompts to conform to the behavior of other family members around the same time [52,53], especially considering that family members received their time-triggered EMAs at approximately the same time as each other.

Studies have used EMA to measure various dietary outcomes, including frequency of food intake, intake of specific types of foods (eg, low glycemic index foods), and energy intake [25]. It has been suggested in a recent systematic review of mobile ecological momentary diet assessment methods that EMA has the potential to be a novel dietary assessment method, both on its own and as a supplement to other mHealth technologies [25]. The use of EMA to assess dietary intake and eating behavior provides some key advantages, namely, the reduction of participant burden and recall bias and the maximization of ecological validity [25]. Taken together with the findings from Dzubur et al [41] and Schembre et al [25], our findings suggest that EMA can be used to sufficiently supplement automatic dietary assessment (ADA) approaches and may be a particularly useful approach for leveraging social relations and maintaining compliance in dyad- and group-based EMA studies.

### Evaluating ADA

Various technologies have been used to passively measure eating activity in naturalistic settings over long periods with minimal user interaction. One of the most popular technologies for assessing eating activity in the field is the wrist-worn smartwatch or accelerometer [23,27]. The performance of automatic, wearable-based, in-field eating detection approaches to date has been reviewed by Bell et al [27]. The smartwatch used in the M2FED study performed on par with other in-field devices, although comparability is difficult owing to the wide and varying metrics used by other papers [27]. Although some wearable devices included in this review performed very well, the duration of the free-living deployment was 1 day (approximately 24 hours) or shorter for more than half of the studies, and another one-third were 1 week in length or shorter [27].

Overall, 3 studies had durations that lasted at least two weeks or longer [34,54,55], 66% (n=2) of which had sample sizes of only 1 participant each. Therefore, the M2FED study is one of the first studies to extensively test the feasibility of deploying an ADA approach for a considerable amount of time (2 weeks) and with a relatively large same size (>50 participants). Part of this success stems from the combined use of mobile devices (for EMA) and smartwatches, which were selected for the M2FED study to maximize long-term usability. Although other technologies have been able to perform better in the field, the usability of these technologies (electromyography electrodes, ear and neck sensors, wearable video cameras, etc) may be lower compared with wrist-worn devices because of the inconvenient location of sensor placement, the potential to interfere with the behavior of participants in real life [56], and the potential intrusiveness or discomfort caused by the sensor [57].

This study also demonstrates that EMA is a feasible tool for collecting ground-truth eating activity and thus evaluating the performance of wearable sensors in the field. Only 2 studies [34,35] included in the review by Bell et al [27] used a novel method for obtaining ground-truth eating activity in the wild, similar to the way EMA was used in the M2FED study. In a study by Ye et al [34], when an eating gesture was automatically detected via a wrist-worn sensor, participants were sent a short message on their smartwatch to confirm or reject in real time whether they were eating. Similarly, in a study by Gomes and Sousa [35], when drinking activity was detected via a wearable sensor, participants were sent an alert on their smartphone and could then confirm or reject whether they were drinking via EMA. Although EMA and similar self-report methods have their own limitations [23,58], they offer the ability to capture and validate ground-truth eating activity near the time of eating, thus improving research scalability and participant acceptability [25].

Another key feature of the M2FED study was the ability to capture intrapersonal (individual) and interpersonal (social) contexts with our combined event- and signal-contingent protocols. A systematic review noted that <7% of EMA studies assessing diet use a combined approach [59]. EMA is a powerful tool that can be used to validate automatically detected eating behavior in the field and to easily collect information about meaningful contexts; however, few studies have used this approach and still rely on paper–pen questionnaires to validate their findings [27].

### Limitations and Strengths

The M2FED study design had notable limitations. First, our method of obtaining ground-truth eating was only deployed via eating event–triggered EMA after an eating event was detected by the smartwatch. Thus, we could only verify true positive and false positive eating events. The M2FED system was not designed to verify true negative or false negative eating events, which limited our ability to calculate common evaluation metrics (ie, accuracy and F_1_-score) and compare our results to other in-field studies described in the literature. Future research can build upon our study by implementing a verification of true negative and false negative eating events, via time-triggered EMA or other methods, to gain a better understanding of the strengths and weaknesses of such an event detection algorithm.

Second, the false positive eating events were self-reported validation, which might be subject to social desirability in underreporting an eating event. This could potentially bias the validity of the results. Third, we encountered various difficulties with the deployed technologies, including smartwatches (ie, limited battery), mobile phones (ie, limited battery and app crashes), and the Wi-Fi router (ie, wireless connection dropped). Although these challenges were anticipated and were addressed in a timely manner on all occasions, some data were lost during the data collection process.

Finally, as the scope of this study only covered in-home eating behavior, we observed relatively few eating event–triggered EMAs per person across the 2-week study (approximately 8 per person). However, the range was very wide, indicating that some participants consumed more meals inside their homes compared with others. Reasons often provided informally by participants included eating all or most meals at school or work, working early or late, traveling for work, and participating in after-school extracurricular activities.

On the other hand, this study also possesses several strengths. First, we recruited a large and ethnically diverse sample of families from Los Angeles. It has been previously noted that the lack of diverse samples in eating-related mHealth and EMA studies is a major limitation of past research [60]. Second, as noted above, the M2FED study facilitated one of the longest in-field deployments found in the literature so far. Most ADA research has been conducted in the laboratory. By deploying in the field, we are able to better understand real-life eating behavior (vs eating behavior in a laboratory) and gain a better understanding of the challenges that arise when deploying wearable sensors outside of the laboratory. Third, as the deployment process was across a 2-year period, we were able to iteratively improve our automatic eating event detection algorithm and then use the newest version in the following deployments. Finally, this study produced momentary measures of theoretical constructs as well as momentary measures of eating behaviors. The theoretical work that we can now contribute would be to understand which constructs influence behavior, which behaviors influence various constructs, and which constructs play no role at all. We can also begin to understand the role of timing in these influences.

### Future Directions

The mHealth field is converging toward the use of a combination of user-friendly devices to assess eating behavior in the wild (eg, mobile phones and wrist-worn devices) [27,31]. Implementing user-friendly technologies for in-field dietary assessment or eating behavior interventions offers at least two substantial advantages—people are generally familiar with them [31] and may be willing to use them for longer periods compared with more intrusive devices. Although early studies experimented with less familiar, often not off-the-shelf technologies (eg, piezoelectric strain gauge sensors), most recent studies have opted for accelerometers and gyroscopes that are embedded within a wrist-worn smartwatch [27]. Furthermore, the combination of a wrist-worn smartwatch to automatically detect eating and a mobile or wearable device to capture ground-truth eating has been featured in a few studies published in the past year [61-63]. This approach is becoming more common, and these types of devices offer advantages for the user (participant) and make the use of mHealth technologies more accessible to nonengineering behavioral researchers. However, a number of related challenges have emerged. Future research will need to address comparability between newer technology-assisted measures and more traditional self-report measures of eating [64] versus other similar technology-assisted measures [27].

These user-friendly technologies also allow for passive measurement or low-effort reporting of various contexts and environments with relative ease. For example, fine-grained real-time GPS data can be scraped from both mobile devices and smartwatches to determine an individual’s location and potentially assess the external influences on behavior [65,66]. Similarly, the social environment can be gleaned from wearable cameras [67], self-report EMA [68], or proximity Bluetooth sensors [69].

The ability to determine one’s context or environment is a necessary component of ecological momentary interventions [70] or just-in-time interventions [71]. These types of intervention designs aim to provide the right amount of support at the right time and in the right context to promote behavior change [71-73]. These types of designs are well-suited for and offer unique opportunities for family-based settings [74]. They offer the ability to intervene in children and adolescents and can be designed to target the behavior of multiple family members at once [74]. As family members share genetic, environmental, and behavioral risks, family units are especially important targets for intervention and prevention [75] and have the potential to halt the intergenerational transmission of obesity and other chronic diseases.

### Conclusions

This paper demonstrates that EMA is a feasible tool to collect ground-truth eating activity and thus evaluate the performance of wearable sensors in the field. The combination of a wrist-worn smartwatch to automatically detect eating and a mobile or wearable device to capture ground-truth eating activity offers key advantages for the user (participant) and makes the use of mHealth technologies more accessible to nonengineering behavioral researchers.

## Abbreviations

ADA: automatic dietary assessment
EMA: ecological momentary assessment
H-t-M: hand-to-mouth
M2FED: Monitoring and Modeling Family Eating Dynamics
mHealth: mobile health
OR: odds ratio

## References

[1] Shim J, Oh K, Kim HC (2014). Dietary assessment methods in epidemiologic studies. Epidemiol Health.

[2] Thompson FE, Subar AF, Loria CM, Reedy JL, Baranowski T (2010). Need for technological innovation in dietary assessment. J Am Diet Assoc.

[3] Magarey A, Watson J, Golley RK, Burrows T, Sutherland R, McNaughton SA, Denney-Wilson E, Campbell K, Collins C (2011). Assessing dietary intake in children and adolescents: considerations and recommendations for obesity research. Int J Pediatr Obes.

[4] Willett W (1998). 24-Hour Dietary Recall and Food Record Methods.

[5] Beaton GH, Burema J, Ritenbaugh C (1997). Errors in the interpretation of dietary assessments. Am J Clin Nutr.

[6] Thompson FE, Kirkpatrick SI, Subar AF, Reedy J, Schap TE, Wilson MM, Krebs-Smith SM (2015). The National Cancer Institute's Dietary Assessment Primer: a resource for diet research. J Acad Nutr Diet.

[7] Livingstone MB, Robson PJ, Wallace JM (2004). Issues in dietary intake assessment of children and adolescents. Br J Nutr.

[8] Westerterp KR, Goris AH (2002). Validity of the assessment of dietary intake: problems of misreporting. Curr Opin Clin Nutr Metab Care.

[9] Althubaiti A (2016). Information bias in health research: definition, pitfalls, and adjustment methods. J Multidiscip Healthc.

[10] Wehling H, Lusher J (2019). People with a body mass index ⩾30 under-report their dietary intake: a systematic review. J Health Psychol.

[11] Novotny JA, Rumpler WV, Riddick H, Hebert JR, Rhodes D, Judd JT, Baer DJ, McDowell M, Briefel R (2003). Personality characteristics as predictors of underreporting of energy intake on 24-hour dietary recall interviews. J Am Diet Assoc.

[12] Willett W (2013). Nutritional Epidemiology.

[13] Nishida C, Uauy R, Kumanyika S, Shetty P (2004). The Joint WHO/FAO Expert Consultation on diet, nutrition and the prevention of chronic diseases: process, product and policy implications. Public Health Nutr.

[14] Wood W, Neal DT (2016). Healthy through habit: interventions for initiating and maintaining health behavior change. Behav Sci Policy.

[15] Neuhouser ML (2019). The importance of healthy dietary patterns in chronic disease prevention. Nutr Res.

[16] Jannasch F, Kröger J, Schulze MB (2017). Dietary patterns and type 2 diabetes: a systematic literature review and meta-analysis of prospective studies. J Nutr.

[17] Higgs S, Thomas J (2016). Social influences on eating. Curr Opin Behav Sci.

[18] Tourlouki E, Matalas AL, Panagiotakos DB (2009). Dietary habits and cardiovascular disease risk in middle-aged and elderly populations: a review of evidence. Clin Interv Aging.

[19] Robinson E, Thomas J, Aveyard P, Higgs S (2014). What everyone else is eating: a systematic review and meta-analysis of the effect of informational eating norms on eating behavior. J Acad Nutr Diet.

[20] Reicks M, Banna J, Cluskey M, Gunther C, Hongu N, Richards R, Topham G, Wong S (2015). Influence of parenting practices on eating behaviors of early adolescents during independent eating occasions: implications for obesity prevention. Nutrients.

[21] Riley WT, Rivera DE, Atienza AA, Nilsen W, Allison SM, Mermelstein R (2011). Health behavior models in the age of mobile interventions: are our theories up to the task?. Transl Behav Med.

[22] Spruijt-Metz D, Hekler E, Saranummi N, Intille S, Korhonen I, Nilsen W, Rivera DE, Spring B, Michie S, Asch DA, Sanna A, Salcedo VT, Kukakfa R, Pavel M (2015). Building new computational models to support health behavior change and maintenance: new opportunities in behavioral research. Transl Behav Med.

[23] Spruijt-Metz D, Wen CK, Bell BM, Intille S, Huang JS, Baranowski T (2018). Advances and controversies in diet and physical activity measurement in youth. Am J Prev Med.

[24] Shiffman S, Stone AA, Hufford MR (2008). Ecological momentary assessment. Annu Rev Clin Psychol.

[25] Schembre SM, Liao Y, O'Connor SG, Hingle MD, Shen S, Hamoy KG, Huh J, Dunton GF, Weiss R, Thomson CA, Boushey CJ (2018). Mobile ecological momentary diet assessment methods for behavioral research: systematic review. JMIR Mhealth Uhealth.

[26] Engel SG, Crosby RD, Thomas G, Bond D, Lavender JM, Mason T, Steffen KJ, Green DD, Wonderlich SA (2016). Ecological momentary assessment in eating disorder and obesity research: a review of the recent literature. Curr Psychiatry Rep.

[27] Bell BM, Alam R, Alshurafa N, Thomaz E, Mondol AS, de la Haye K, Stankovic JA, Lach J, Spruijt-Metz D (2020). Automatic, wearable-based, in-field eating detection approaches for public health research: a scoping review. NPJ Digit Med.

[28] Spruijt-Metz D, de la Haye K, Lach J, Stankovic J (2016). M2FED: Monitoring and Modeling Family Eating Dynamics. Proceedings of the 14th ACM Conference on Embedded Network Sensor Systems CD-ROM.

[29] Vu T, Lin F, Alshurafa N, Xu W (2017). Wearable food intake monitoring technologies: a comprehensive review. Computers.

[30] Hassannejad H, Matrella G, Ciampolini P, De Munari I, Mordonini M, Cagnoni S (2017). Automatic diet monitoring: a review of computer vision and wearable sensor-based methods. Int J Food Sci Nutr.

[31] Heydarian H, Adam M, Burrows T, Collins C, Rollo ME (2019). Assessing eating behaviour using upper limb mounted motion sensors: a systematic review. Nutrients.

[32] Boushey CJ, Spoden M, Zhu FM, Delp EJ, Kerr DA (2016). New mobile methods for dietary assessment: review of image-assisted and image-based dietary assessment methods. Proc Nutr Soc.

[33] Gemming L, Utter J, Ni Mhurchu C (2015). Image-assisted dietary assessment: a systematic review of the evidence. J Acad Nutr Diet.

[34] Ye X, Chen G, Gao Y, Wang H, Cao Y (2016). Assisting food journaling with automatic eating detection. Proceedings of the 2016 CHI Conference Extended Abstracts on Human Factors in Computing Systems.

[35] Gomes D, Sousa I (2019). Real-time drink trigger detection in free-living conditions using inertial sensors. Sensors.

[36] Wen CK, Schneider S, Stone AA, Spruijt-Metz D (2017). Compliance with mobile ecological momentary assessment protocols in children and adolescents: a systematic review and meta-analysis. J Med Internet Res.

[37] Liao Y, Skelton K, Dunton G, Bruening M (2016). A systematic review of methods and procedures used in ecological momentary assessments of diet and physical activity research in youth: an adapted STROBE checklist for reporting EMA studies (CREMAS). J Med Internet Res.

[38] Maher JP, Rebar AL, Dunton GF (2018). Ecological momentary assessment is a feasible and valid methodological tool to measure older adults' physical activity and sedentary behavior. Front Psychol.

[39] Heron KE, Everhart RS, McHale SM, Smyth JM (2017). Using mobile-technology-based ecological momentary assessment (EMA) methods with youth: a systematic review and recommendations. J Pediatr Psychol.

[40] Nam S, Whittemore R, Vlahov D, Dunton G (2019). 824-P: ecological momentary assessment of diabetes self-management: a systematic review of methods and procedures. Diabetes.

[41] Dzubur E, Huh J, Maher JP, Intille SS, Dunton GF (2018). Response patterns and intra-dyadic factors related to compliance with ecological momentary assessment among mothers and children. Transl Behav Med.

[42] Rajkumar R, Lee I, Sha L, Stankovic J (2010). Cyber-physical systems: the next computing revolution. Proceedings of the 47th Design Automation Conference.

[43] Mondol MA, Bell B, Ma M, Alam R, Emi I, Preum S, de la Haye K, Spruijt-Metz D, Lach J, Stankovic JA (2020). MFED: a system for monitoring family eating dynamics. arXiv.

[44] Laurent J, Catanzaro SJ, Joiner TE, Rudolph KD, Potter KI, Lambert S, Osborne L, Gathright T (1999). A measure of positive and negative affect for children: scale development and preliminary validation. Psychol Assess.

[45] Cohen S, Kamarck T, Mermelstein R (1983). A global measure of perceived stress. J Health Soc Behav.

[46] Forrest CB, Ravens-Sieberer U, Devine J, Becker BD, Teneralli RE, Moon J, Carle AC, Tucker CA, Bevans KB (2018). Development and evaluation of the PROMIS pediatric positive affect item bank, child-report and parent-proxy editions. J Happiness Stud.

[47] Terry PC, Lane AM, Lane HJ, Keohane L (1999). Development and validation of a mood measure for adolescents. J Sports Sci.

[48] Cardello AV, Schutz HG, Lesher LL, Merrill E (2005). Development and testing of a labeled magnitude scale of perceived satiety. Appetite.

[49] Framson C, Kristal AR, Schenk JM, Littman AJ, Zeliadt S, Benitez D (2009). Development and validation of the mindful eating questionnaire. J Am Diet Assoc.

[50] Ma M, Alam R, Bell B, de la Haye K, Spruijt-Metz D, Lach J, Stankovic J (2017). M^2G: a monitor of monitoring systems with ground truth validation features for research-oriented residential applications. Proceedings of the IEEE 14th International Conference on Mobile Ad Hoc and Sensor Systems (MASS).

[51] Grolemund G, Wickham H (2011). Dates and times made easy with lubridate. J Stat Soft.

[52] Cialdini RB, Goldstein NJ (2004). Social influence: compliance and conformity. Annu Rev Psychol.

[53] Cialdini RB, Trost MR, Gilbert GT, Fiske ST, Lindzey G (1998). Social influence: social norms, conformity and compliance. The Handbook of Social Psychology.

[54] Navarathna P, Bequette B, Cameron F (2018). Wearable device based activity recognition and prediction for improved feedforward control. Proceedings of the Annual American Control Conference (ACC).

[55] Thomaz E, Essa I, Abowd GD (2015). A practical approach for recognizing eating moments with wrist-mounted inertial sensing. Proceedings of the 2015 ACM International Joint Conference on Pervasive and Ubiquitous Computing.

[56] Rast FM, Labruyère R (2020). Systematic review on the application of wearable inertial sensors to quantify everyday life motor activity in people with mobility impairments. J Neuroeng Rehabil.

[57] Fontana JM, Farooq M, Sazonov E, Sazonov E (2021). Chapter 20 - Detection and characterization of food intake by wearable sensors. Wearable Sensors (Second Edition).

[58] McClung HL, Ptomey LT, Shook RP, Aggarwal A, Gorczyca AM, Sazonov ES, Becofsky K, Weiss R, Das SK (2018). Dietary intake and physical activity assessment: current tools, techniques, and technologies for use in adult populations. Am J Prev Med.

[59] Maugeri A, Barchitta M (2019). A systematic review of ecological momentary assessment of diet: implications and perspectives for nutritional epidemiology. Nutrients.

[60] Smith KE, Juarascio A (2019). From Ecological Momentary Assessment (EMA) to Ecological Momentary Intervention (EMI): past and future directions for ambulatory assessment and interventions in eating disorders. Curr Psychiatry Rep.

[61] Morshed MB, Kulkarni SS, Li R, Saha K, Roper LG, Nachman L, Lu H, Mirabella L, Srivastava S, De Choudhury M, de Barbaro K, Ploetz T, Abowd GD (2020). A real-time eating detection system for capturing eating moments and triggering ecological momentary assessments to obtain further context: system development and validation study. JMIR Mhealth Uhealth.

[62] Goldstein SP, Hoover A, Evans EW, Thomas JG (2021). Combining ecological momentary assessment, wrist-based eating detection, and dietary assessment to characterize dietary lapse: A multi-method study protocol. Digit Health.

[63] Sen S, Subbaraju V, Misra A, Balan R, Lee Y (2020). Annapurna: an automated smartwatch-based eating detection and food journaling system. Perv Mobile Comput.

[64] Fowler LA, Grammer AC, Staiano AE, Fitzsimmons-Craft EE, Chen L, Yaeger LH, Wilfley DE (2021). Harnessing technological solutions for childhood obesity prevention and treatment: a systematic review and meta-analysis of current applications. Int J Obes (Lond).

[65] Yang J, Wang J, Nakandala S, Kumar A, Jankowska M (2019). Predicting eating events in free living individuals. Proceedings of the 15th International Conference on eScience (eScience).

[66] Cetateanu A, Jones A (2016). How can GPS technology help us better understand exposure to the food environment? A systematic review. SSM Popul Health.

[67] Gemming L, Doherty A, Utter J, Shields E, Ni Mhurchu C (2015). The use of a wearable camera to capture and categorise the environmental and social context of self-identified eating episodes. Appetite.

[68] O'Connor SG, Habre R, Bastain TM, Toledo-Corral CM, Gilliland FD, Eckel SP, Cabison J, Naya CH, Farzan SF, Chu D, Chavez TA, Breton CV, Dunton GF (2019). Within-subject effects of environmental and social stressors on pre- and post-partum obesity-related biobehavioral responses in low-income Hispanic women: protocol of an intensive longitudinal study. BMC Public Health.

[69] Mundnich K, Booth BM, L'Hommedieu M, Feng T, Girault B, L'Hommedieu J, Wildman M, Skaaden S, Nadarajan A, Villatte JL, Falk TH, Lerman K, Ferrara E, Narayanan S (2020). TILES-2018, a longitudinal physiologic and behavioral data set of hospital workers. Sci Data.

[70] Heron KE, Smyth JM (2010). Ecological momentary interventions: incorporating mobile technology into psychosocial and health behaviour treatments. Br J Health Psychol.

[71] Spruijt-Metz D, Nilsen W (2014). Dynamic models of behavior for just-in-time adaptive interventions. IEEE Pervasive Comput.

[72] Nahum-Shani I, Hekler EB, Spruijt-Metz D (2015). Building health behavior models to guide the development of just-in-time adaptive interventions: a pragmatic framework. Health Psychol.

[73] Nahum-Shani I, Smith SN, Spring BJ, Collins LM, Witkiewitz K, Tewari A, Murphy SA (2016). Just-in-Time Adaptive Interventions (JITAIs) in mobile health: key components and design principles for ongoing health behavior support. Ann Behav Med.

[74] Heron KE, Miadich SA, Everhart RS, Smyth JM, Fiese BH, Celano M, Deater-Deckard K, Jouriles EN, Whisman MA (2019). Ecological momentary assessment and related intensive longitudinal designs in family and couples research. APA Handbook of Contemporary Family Psychology: Foundations, Methods, and Contemporary Issues Across the Lifespan.

[75] Kral TV, Rauh EM (2010). Eating behaviors of children in the context of their family environment. Physiol Behav.

